# The protective effect of luteolin on the depression-related dry eye disorder through Sirt1/NF-κB/NLRP3 pathway

**DOI:** 10.18632/aging.204479

**Published:** 2023-01-11

**Authors:** Mingxia Xie, Hanqing Wang, Tiantian Gao, Jun Peng, Pan Meng, Xi Zhang, Dongwei Guo, Guangya Liu, Jian Shi, Qinghua Peng

**Affiliations:** 1 College of Traditional Chinese Medicine, Hunan University of Chinese Medicine, Changsha, Hunan 410007, P.R. China; 2 College of Pharmacy, Ningxia Medical University, Yinchuan, Ningxia 750004, P.R. China; 3 College of Clinical Medicine, Hunan University of Chinese Medicine, Changsha, Hunan 410007, P.R. China; 4 The First Hospital of Hunan University of Chinese Medicine, Changsha, Hunan 410007, P.R. China; 5 National Key Laboratory Cultivation Base of Chinese Medicinal Powder and Innovative Medicinal Jointly Established by Province and Ministry, Hunan University of Chinese Medicine, Changsha, Hunan 410007, P.R. China

**Keywords:** luteolin, depression, CUMS, dry eye, Sirt1/NF-κB/NLRP3

## Abstract

Luteolin has been reported to exhibit therapeutic effect on depressive-like behaviors in mice. Nevertheless, the therapeutic effect of luteolin on the depression-related dry eye disorder remains inconclusive. In this study, C57 mice were subjected to chronic unpredictable mild stress in a dry environment (relative humidity in the cage <40%). The behavioral test and phenol red cotton thread test were employed to select the mice with both dry eye and depression-like behavior. The mechanism of luteolin on depression-related dry eye disorder was assessed by the Sirt1 selective inhibitor EX-527. Luteolin alleviated depressive-like behaviors induced by CUMS, increased tear secretion and restored corneal defects in mice. The secretions of pro-inflammatory factors IL-1β, IL-6, IL-18 and TNF-α were decreased in hippocampi and corneal tissues by Luteolin treatment. Luteolin treatment up-regulated Sirt1 expression and down-regulated Ac-NF-κB, NLRP3, Ac-Caspase-1, GSDMD-N, Cleaved IL-1β, and Cleaved IL-18 expressions. In addition, the selective inhibition of Sirt1 could weaken the therapeutic effect of luteolin on depression-related dry eye disorder. The beneficial effect of luteolin through Sirt1/NF-κB/NLRP3 signaling pathway might be a therapeutic strategy for the depression-related dry eye disorder.

## INTRODUCTION

Major depressive disorder (MDD) is an emotional disorder which affects cognitive, motivational, and physiological function [1]. It is usually characterized by persistent low mood and/or inability to experience pleasure during daily life and work [2]. As a multifactor disease of tear and ocular surface, dry eye disease can be caused by a variety of factors, such as collagen disease, Sjogren's syndrome, malnutrition, graft-versus-host disease and drugs, etc. [3]. Dry eye disease (DED) affects millions of people worldwide and its prevalence ranges from 5% to 50% [4]. The main symptoms of DED are burning or tingling, foreign body sensation, photophobia and blurred vision. Although the symptoms of dry eye disease are rarely particularly severe, chronic ocular surface irritation and visual impairment directly reduce the life quality of patients with dry eye [5].

More and more people around the world are suffering from two or more long-term diseases at the same time, known as multimorbidity. In the UK, the proportion of people with multiple diseases is expected to grow from 54% in 2015 to 68% in 2035 [6]. Concurrently, the relationship between dry eye and mental disorders is of increasing interest [7]. Dry eye symptoms and mood symptoms may influence each other. It is reported that depression may be a factor aggravating dry eye. The depression led to a nearly twofold increase in the occurrence of dry eye complication [8]. Another study proposed that the patients with depression and posttraumatic stress disorder (PTSD) would have more dry eye symptoms [9]. Thus, we assumed that the attenuation of depressive like behavior was beneficial for the treatment of depression-related dry eye disorder.

DED is an ocular surface immune inflammatory disease in which instability tear film and high osmotic pressure cause pathogenic responses in the cornea and conjunctiva [10]. Due to the release of damage-associated molecules and more inflammatory factors during tissue injury [11], silencing information modulator related enzyme1 (Sirt1) is an NAD+-dependent deacetylase controlling DNA repairment, transcriptional recombination, stress resistance, oxidative stress, inflammation, and apoptosis [12–14]. Inflammation is a key factor initiating and amplifying pathological progression of depression. Inhibiting inflammation may provide benefits to the overall treatment of diverse depression caused by early life trauma, acute stress responses, microbiome alterations and genetic factors. The transcription factor NF-κB drives the activation of NLRP3 inflammasome [15]. Once activated, the NLRP3 inflammasome assembles and leads to diverse changes including caspase1-mediated proteolytic activation of interleukin-1β (IL-1β) family cytokines, the induction of pyroptosis [16, 17], and the release of pro-inflammatory factor. It was believed that the acetylation of NF-κB in the inflammatory response was controlled by Sirt1 [15].

Luteolin (Lut), a common natural polyphenolic flavonoid compounds in edible plants, protects plant cells from harmful external factors [18]. In various models of inflammatory and neurodegenerative diseases, it was demonstrated to exhibit beneficial effects including cytoprotective, neuroprotective, and neurogenic activities [19–21]. Meanwhile, luteolin is lipophilic and can freely cross the blood-brain barrier [22]. It was described that luteolin treatment might alter some signaling pathways associated with depression by regulating the decision of NSCs fate. Luteolin had a restorative effect on neural stem cells (NSCs) signaling pathways to overcome the damage caused by LPS injection [19]. Whether luteolin exerted attenuated activity on depression-accompanied dry eye disease remained elusive. Therefore, in this work, depression-like behaviors were induced by chronic unpredictable mild stress (CUMS) paradigm, and we created a dry environment to make mice appear dry eye state. It was aimed to explore the efficacy of luteolin in a mouse model of dry eye comorbid depression and its molecular mechanism via Sirt1/NF-κB/NLRP3 signaling pathway.

## RESULTS

### Luteolin alleviated depression-like behaviors and dry eye symptoms in a model of depression-related dry eye disorder

**Figure 1:**
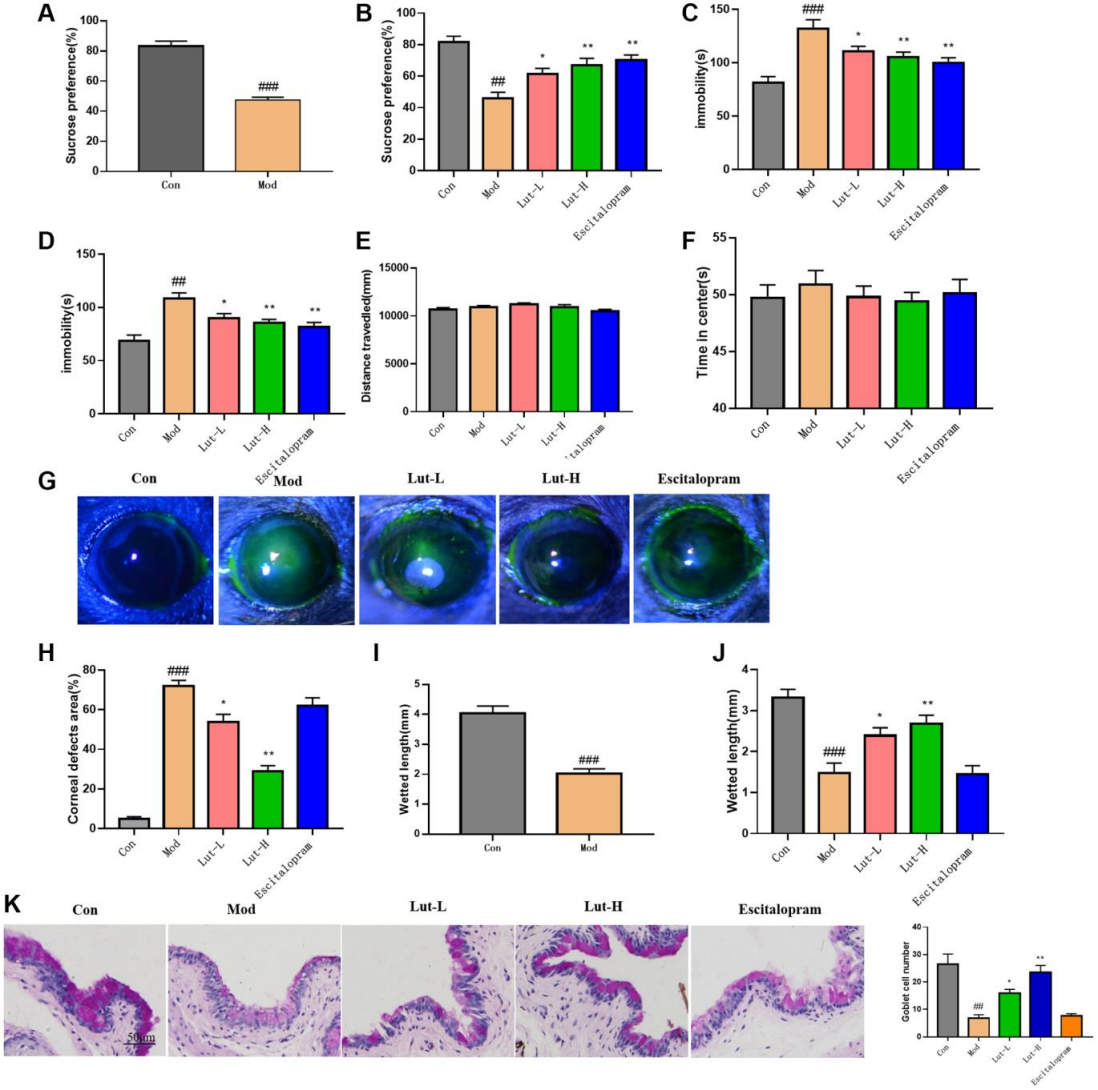
**Luteolin improved depressive behavior and dry eye symptoms in DEDC mice.** (**A**, **B**) SPT, (**C**) TST, (**D**) FST, and (**E**, **F**) OFT. (**G**) Corneal epithelial defect fluorescence representative images of pyruvate sodium staining (**H**) Quantitative determination of corneal defects in mice (**I**, **J**) Phenol red line assay showing tear volume (**K**) The effect of luteolin on goblet cells in xerophthalmia and CUMS mice. Scale bar is 50 μm. Data analysis was performed with Mean ± SEM. ^#^*p* < 0.05, ^##^*p* < 0.01 compared with blank group; ^*^p < 0.05, ^**^p < 0.01 compared with model group, *n* = 9.

To explore whether luteolin had an attenuated effect on CUMS-induced depression-like behaviors in mice, we performed SPT, OFT, TST and FST behavioral experiments. In the SPT experiment, the intake of sucrose in the CUMS-induced mice was significantly lower than that in control group, indicating that the CUMS-induced mice lacked pleasure (Figure 1A). While in the luteolin (5 mg/kg) (*p* < 0.05), luteolin (10 mg/kg) (*p* < 0.01), and escitalopram (*p* < 0.01) treatment groups, the preference for sucrose was significantly increased (Figure 1B). In TST, the immobility time of the mice in the model group was significantly longer than that in control group. Luteolin (10 mg/kg) administration (*p* < 0.01) could significantly reverse this change, which was similar with that of the positive drug escitalopram (*p* < 0.01) (Figure 1C). In FST, compared with CUMS group, luteolin (10 mg/kg) (*p* < 0.01), luteolin (5 mg/kg) (*p* < 0.05) significantly shortened the immobility time of mice (Figure 1D). CUMS stimulation did not significantly affect the distance and time in center zone compared with those of control group. Compared with the control group, CUMS stimulation had no effect on the movement distance and the time of staying in center zone Luteolin (5 mg/kg), luteolin (10 mg/kg), escitalopram treatment group compared with the depression-related dry eye disorder group also had no distinct difference. OFT data showed that luteolin did not affect the motor activity of mice. (Figure 1E, 1F).

Besides, we also tested the effect of luteolin on dry eye symptoms. Sodium fluorescein is commonly used to measure the areas of corneal surface epithelial cells. Depression-related dry eye disorder group showed severe corneal impairment and ocular surface epithelium damage compared with those of control group. Luteolin (5 mg/kg) (*p* < 0.05) and luteolin (10 mg/kg) (*p* < 0.01) significantly attenuated corneal damage in the mice of depression-related dry eye disorder group, respectively (Figure 1G, 1H). Next, we used phenol red cotton thread to measure the tear secretion of mice [23]. Compared with the control group, the tear secretion of mice in the depression-related dry eye disease group was significantly reduced, but it was immensely increased in luteolin (5 mg/kg) group (*p* < 0.05) and luteolin (10 mg/kg) group (*p* < 0.01). (Figure 1I, 1J). In addition, the reduction in goblet cell density was positively associated with disease severity [10]. Conjunctival PAS staining showed that compared with the control group, conjunctival goblet cells in depression related dry eye disease group were significantly reduced (*p* < 0.01). The number of conjunctival goblet cells in the luteolin (5 mg/kg) group increased significantly (*p* < 0.05), and the luteolin (10 mg/kg) group had a better therapeutic effect (*p* < 0.01). Escitalopram, a positive drug for depression-like symptoms, did not significantly influence DED symptoms in mice. (Figure 1K).

### The effect of luteolin on the inflammatory response in depression-related dry eye disorder mice

**Figure 2:**
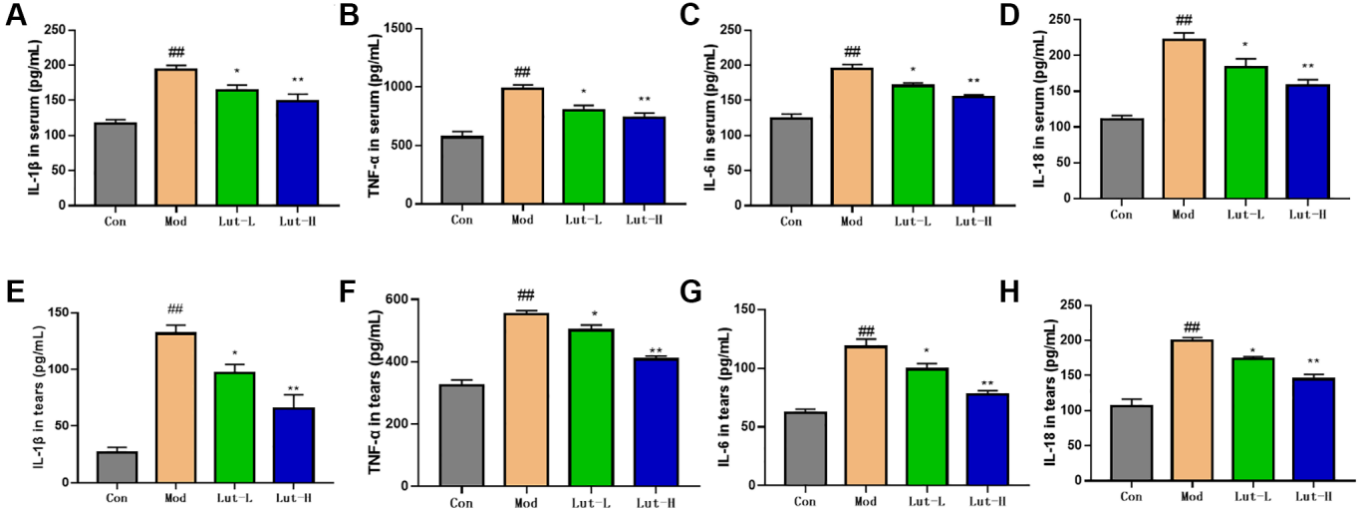
**The regulation of IL-1β, TNF-α, IL-18, and IL-6 in the serum and tears of mice with dry eye and CUMS mice.** (**A**, **B**), (**C**, **D**) are the levels of IL-1β, TNF-α, IL-18, and IL-6 in serum. (**E**–**H**) are the contents of IL-1β, TNF-α, IL-18, and IL-6 in tear fluid. Data analysis was performed with Mean ± SEM. ^##^*p* < 0.01 compared with blank group; ^*^p < 0.05, ^**^p < 0.01 compared with model group, *n* = 9.

Previous studies displayed that the development of dry eye disease and depression was accompanied by inflammation in rodents [24] and humans [25]. The related pro-inflammatory factors IL-1β, IL-6, TNF-α and IL-18 were detected in mouse serum using enzyme-linked immunosorbent assay (ELISA). Compared with control group, the pro-inflammatory factors in the tears of model mice were significantly increased (*p* < 0.01). After the treatment with luteolin (5 mg/kg) (*p* < 0.05) and luteolin (10 mg/kg) (*p* < 0.01), the serum levels of pro-inflammatory factors IL-1β, TNF-α, IL-6 and IL-18 were significantly higher than those in the control group. The same trend was also shown for the above pro-inflammatory factors in tears (Figure 2). The results showed that luteolin could reverse and stabilize the elevated pro-inflammatory factors in mice with the depression-related dry eye disorder, and alleviate the inflammatory changes in mice.

### The effects of luteolin on Sirt1/NF-κB/NLRP3 signaling pathway

**Figure 3:**
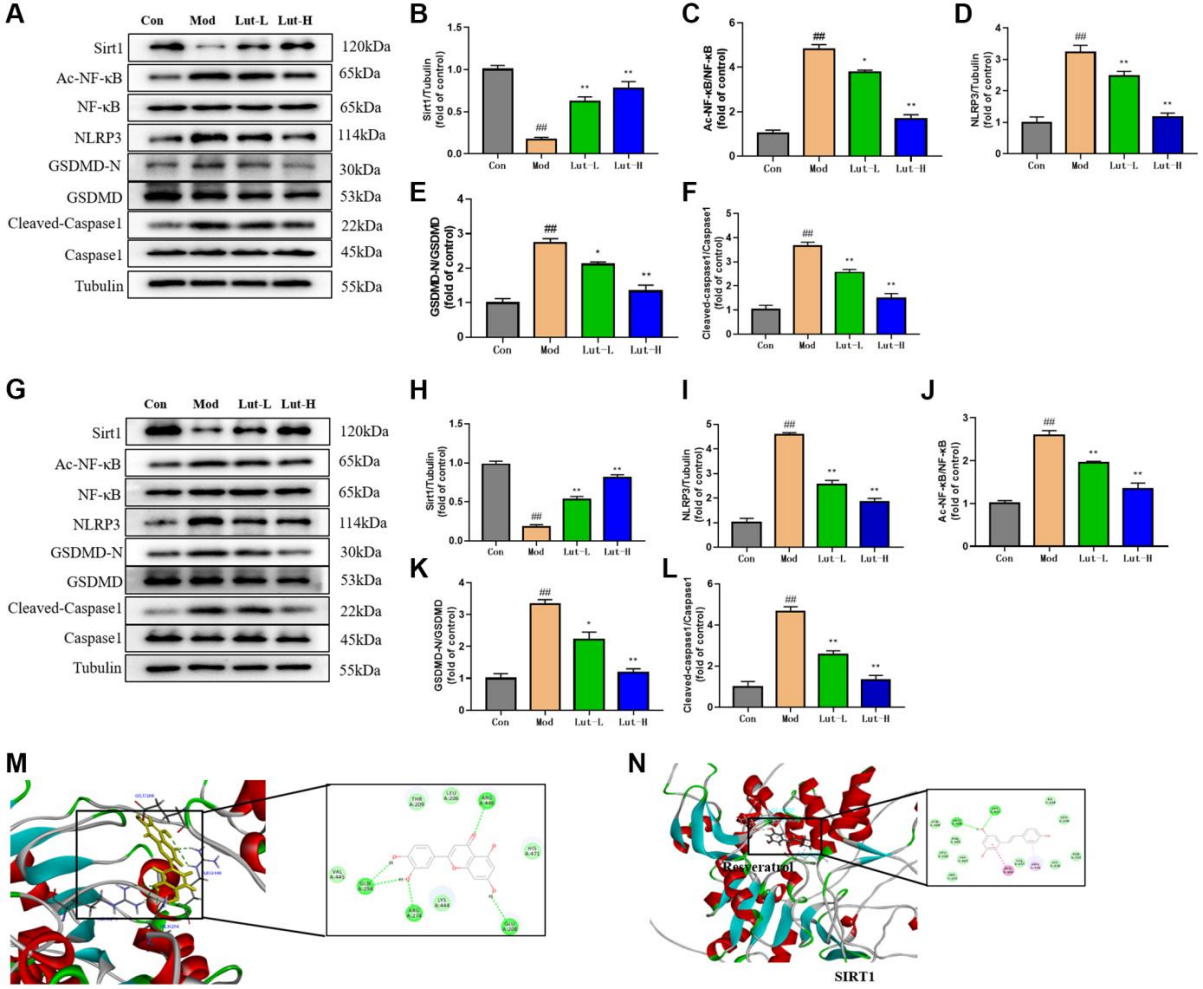
**Luteolin could regulate the expressions of Sirt1, Nlrp3, AC-NF -κB, NF-κB, Nlrp3, GSDMD-N and GSDMD proteins in hippocampi and tears of mice.** (**A**–**F**) Western Blot was used to detect the effects of luteolin on protein bands of Sirt1, Nlrp3, AC-NF-κB, NF-κB, GSDMD-N and GSDMD in the hippocampus of mice. (**G**–**L**) Western Blot analysis of the effects of luteolin on protein bands of Sirt1, Nlrp3, AC-NF -κB, NF-κB, GSDMD-N and GSDMD in mice cornea. (**M**) Molecular docking of sitrt1 and luteolin in discovery studio. (**N**) Molecular docking of sirt1 and Resveratrol by discovery studio. Data were analyzed by mean ± scanning electron microscopy. Compared with blank group, ^##^*P* < 0.01; Comparison with model group ^*^P < 0.05, ^**^P < 0.01, *n* = 9.

The activity of Sirt1 in mouse hippocampal dentate gyrus was decreased after chronic stress. Additionally, inhibition of hippocampal Sirt1 function in mice on the basis of genetics and pharmacology can lead to the increase of depression-like behaviors [26]. Due to the reversal of depressive behaviors and dry eye symptoms in mice, we speculated that luteolin might act on Sirt1 protein to treat depression-related dry eye disorder in mice. To clarify the therapeutic effect of luteolin on CUMS-induced depression, we used Western Blot to detect the protein expression levels of Sirt1, NLRP3, Cleaved-caspase1 and Caspase1 in the mouse hippocampus (Figure 3A). The results showed that compared with the control group, the level of Sirt1 protein in the hippocampus of CUMS-induced mice was significantly decreased (*P* < 0.01). Both the luteolin (5 mg/kg) treatment group (*p* < 0.05) and the luteolin (10 mg/kg) (*P* < 0.01) treatment group significantly increased Sirt1 protein levels (Figure 3B). The protein expression levels of Ac-NF-κB, NF-κB, NLRP3, GSDMD-N, GSDMD, Cleaved-Caspase1 and Caspase1 were significantly up-regulated after CUMS stimulation (*p* < 0.01). The treatment with luteolin (5 mg/kg) and luteolin (10 mg/kg) could significantly reverse the down-regulations of protein expression of Ac-NF-κB, GSDMD-N and GSDMD (*p* < 0.01). In addition, NLRP3, Cleaved-Caspase1 and Caspase1 protein expression levels were remarkably down-regulated after luteolin (5 mg/kg) (*p* < 0.05) and luteolin (10 mg/kg) (*p* < 0.01) treatments (Figure 3C–3F). Similarly, we also detected the above proteins in mouse cornea tissue, showing the same change trend as in mouse hippocampus (Figure 3G–3L).

As previously described, we performed further validation of the interaction between luteolin and Sirt1 protein by using molecular docking. We conducted molecular docking between Sirt1 protein and luteolin through Discovery Studio software, and the CDOCKER ENERGY of the two docking was −24.4965 kcal/mol, indicating that Sirt1 protein was related to luteolin and relatively closely bound. (Figure 3M). Luteolin might combine with Sirt1 at GLU208, ARG274, GLN294 and ARG446 via hydrogen bond. We hypothesized that luteolin played a role as a Sirt1 activator in the treatment of depressive-like behavior in mice. Surprisingly, the CDOCKER ENERGY of Sirt1 activator resveratrol and Sirt1 protein was −20.3636 kcal/mol, indicating that luteolin was more tightly connected to Sirt1 than resveratrol. However, the specific action relationship between luteolin and Sirt1 needs to be further investigated (Figure 3N).

### The effects of luteolin on microglia

**Figure 4:**
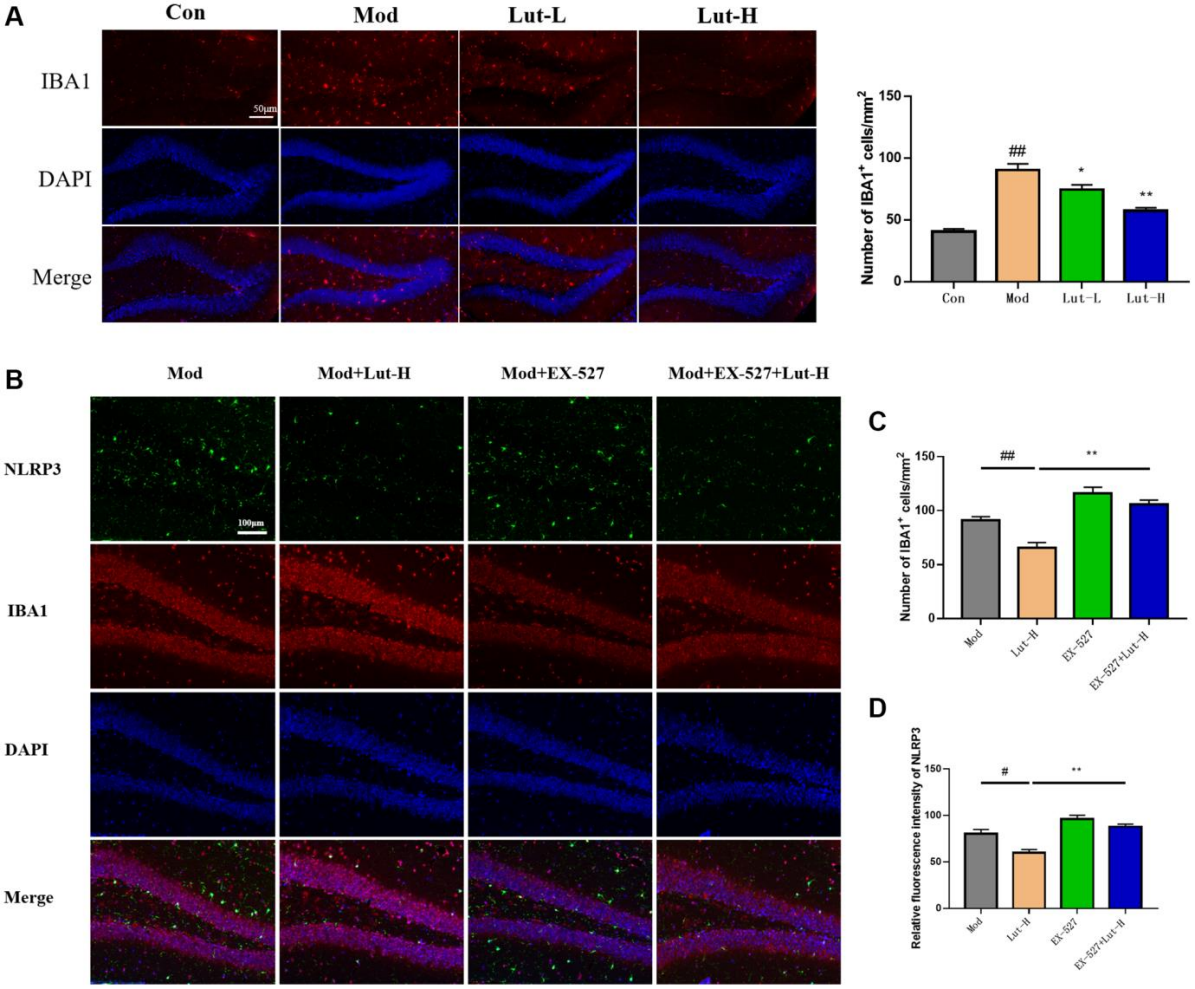
**Effects of immunofluorescence on microglia in mice.** (**A**) Effects of luteolin on microglia in different doses were determined by immunofluorescence. (**B**) Determination of microglia in the hippocampal area of mice after EX-527. (**C**) Effect of EX-527 on microglia activation. (**D**) Relative fluorescence intensity of Nlrp3. Data were analyzed by mean ± SEM. Compared with blank group, ^##^*P* < 0.01; Comparison with model group ^*^P < 0.05, ^**^P < 0.01, *n* = 9.

Iba1, a calcium-binding protein, is only expressed in microglia in the central nervous system (CNS) and is upregulated upon microglia activation [27]. Therefore, Iba1 immunofluorescence staining was used to detect the activation state of microglia. Considering the critical role of microglia in neuroinflammation, we examined microglia in mouse hippocampus by immunofluorescence. CUMS exposure significantly increased hippocampal microglia activation compared to controls (*p* < 0.01). The treatments with luteolin (5 mg/kg) (*p* < 0.05) and luteolin (10 mg/kg) (*p* < 0.01) hampered the upregulation of microglia induced by CUMS exposure (Figure 4A).

In addition, the Sirt1 protein inhibitor EX-527 was further used to explore the role of Sirt1 in CUMS-exposed mice. The effect of luteolin (10 mg/kg) on microglia activation was undoubtedly abolished by EX-527, which was positive for Iba1. The number of cells increased, suggesting that luteolin reduces microglia activation by activating cells through the Sirt1 protein (Figure 4B, 4C).

### EX-527 blocked luteolin mediated treatment of depression-like behaviors in mice

**Figure 5:**
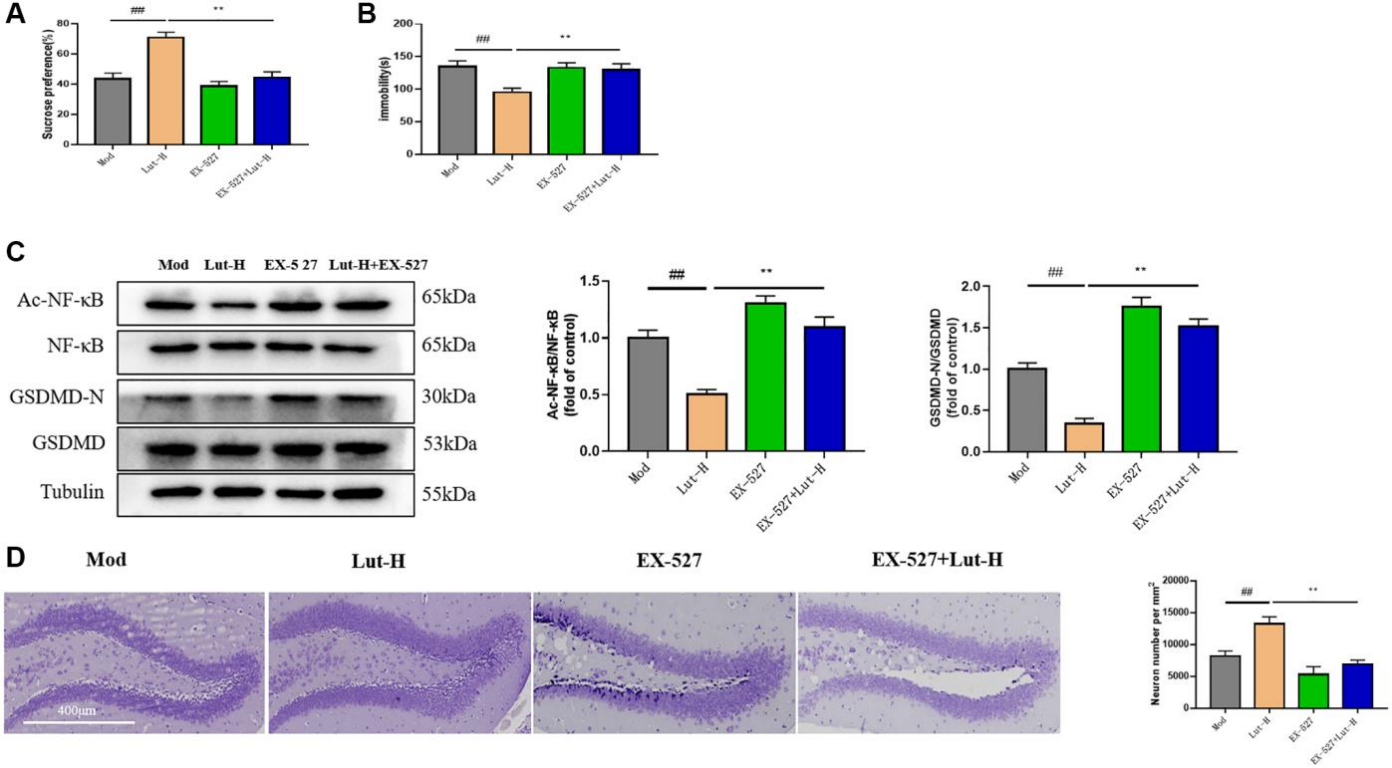
**Role of Sirt1 in the reduction of CUMS-induced depressive behavior by luteolin.** (**A**) Sucrose preference rate of mice was determined by SPT. (**B**) The immobility time of mice was measured by TST. (**C**) Western Blot analysis of the expression of AC-NF-κB, NF-κB, GSDMD-N and GSDMD protein in hippocampal tissue. (**D**) Nisi staining of mice treated with EX-527. Data were analyzed by means ± SEM. Compared with blank group, ^##^*P* < 0.01; Comparison with model group ^*^P < 0.05, ^**^P < 0.01, *n* = 9.

To further investigate the *in vivo* role of Sirt1 signaling in the antidepressant mechanism of luteolin, we further investigated the mechanism of luteolin against depression-related dry eye disorder using the SIRT1 selective inhibitor EX-527. As shown in the Figure 5A, Sirt1 inhibitor EX-527 inhibited luteolin (10 mg/kg) from improving sucrose preference in mice in SPT (*p* < 0.01). Unsurprisingly, EX-527 also inhibited the anti-depressive like effect of luteolin (10 mg/kg) in TST. The immobility time of mice was significantly longer when EX-527 and luteolin (10 mg/kg) were used together than when luteolin (10 mg/kg) was used alone (*p* < 0.01) (Figure 5B). These results revealed that the mechanism of luteolin against pleasure loss and behavioral despair in depressed mice depends on Sirt1 signaling. Give the above, after applying EX-527, we used Western Blot to detect the protein expressions of Ac-NF-κB, NF-κB, GSDMD-N and GSDMD in hippocampus. EX-527 resisted the decreased expression of Ac-NF-κB, NF-κB, GSDMD-N and GSDMD protein in hippocampus treated with luteolin (10 mg/kg) compared to the group treated with luteolin (10 mg/kg) alone (Figure 5C).

Immunofluorescence showed that EX-527 inhibited the abolition of the relative fluorescence intensity of NLRP3 by luteolin (10 mg/kg) compared with luteolin (10 mg/kg) alone in the dry eye and depressive comorbid groups (*p* < 0.01) (Figure 4D).

Histological changed in the DG region of mice exposed to CUMS were visualized using Nissl staining. CUMS stimulation darkened the staining of positive cells, whereas after luteolin (10 mg/kg) treatment, nuclei and abundant Nissl vesicles could be seen. As shown in the Figure 5D, luteolin (10 mg/kg) increased the number of neurons in Nissl body, but EX-527 reversed this change, suggesting that luteolin had a protective role in the damage of cortical neurons induced by CUMS exposed mice protection, and Sirt1 was involved in this process.

### EX-527 blocks the effect of luteolin in treating symptoms of dry eye disease in mice

**Figure 6:**
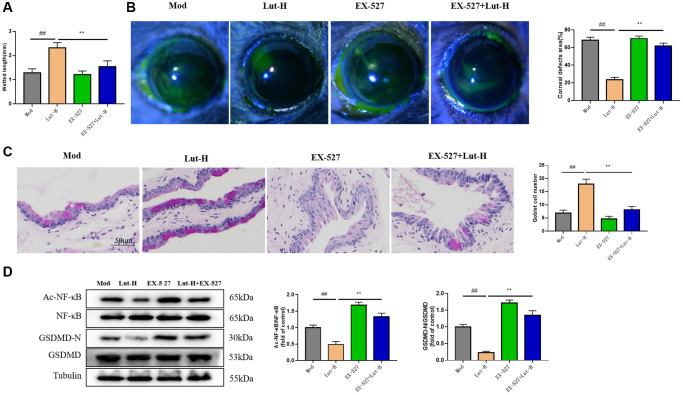
**Effects of Sirt1 on luteolin in alleviating dry eye symptoms in DEDC mice.** (**A**) Phenol red cotton thread testing mouse tears. (**B**) Sodium fluorescein staining. (**C**) Goblet cell staining. (**D**) Western blotting detects protein expression of AC-NF-κB, NF-κB and GSDMD-N, GSDMD in tear fluid. Data analysis was performed with mean ± SEM. ^##^*p* < 0.01 compared with blank group; ^*^p < 0.05, ^**^p < 0.01 compared with model group, *n* = 9.

To further elucidate the role of Sirt1 protein and dry eye-like symptoms in mice, we tested the tear secretion of mice in the dry eye and depression comorbidity group after the application of EX-527. EX-527 significantly reversed the increased tear secretion in the luteolin (10 mg/kg)-treated group compared with the depression and dry eye comorbid groups treated with luteolin (10 mg/kg) alone (*P* < 0.01) (Figure 6A). In addition, EX-527 and luteolin (10 mg/kg) inhibited the repairing effect of luteolin (10 mg/kg) on corneal defect (Figure 6B).

In addition, PAS staining of the conjunctival epithelium showed that EX-527 significantly inhibited the protective effect of luteolin on goblet cells of the conjunctival epithelium (Figure 6C).

Besides, the corneal Ac-NF-κB, NF-κB, GSDMD-N and GSDMD protein expressions were detected. Not surprisingly, compared with luteolin alone (10 mg/kg), the simultaneous use of EX-527 and luteolin (10 mg/kg) could abrogate the effect of luteolin on Ac-NF-κB, NF-κB, GSDMD-N and GSDMD protein (Figure 6D).

These results suggested that Sirt1 played an important role in the treatment of dry eye symptoms with luteolin (10 mg/kg).

## DISCUSSION

An increasing number of people around the world are suffering from two or more long-term conditions at the same time, known as co-morbidities [6]. One is to take different drugs to treat each disease individually, one by one, but this approach has the disadvantage that the combination of multiple drugs may have a negative impact on the patient [28]. The second is to select drugs with a common pharmacological basis, which may have a therapeutic effect on each disease in the co-morbidities, just to make up for the shortcomings of the former approach and reduce the risk of the combination of drugs for the patient [6].

According to the current study, a positive correlation between depression and dry eye has been found [5, 8, 9, 29]. Dry eye is defined as a multifactorial disease of the ocular surface characterized by loss of homeostasis within the tear film. DED is associated with ocular symptoms which are characterized by instable tear film and high osmotic pressure. Ocular surface inflammation and neurosensory abnormality are also critical for this etiology [30]. Specific environments and certain medications (including over-the-counter medications, such as antihistamines) may trigger dry eye. Ocular surface inflammation is an important pathogenesis factor of dry eye. Reducing inflammation may be a treatment method for dry eye [31]. Topical application of TNF-α stimulated gene 6 (Tsg-6) protein is as effective as eye drops of cyclosporine eye milk in the treatment of inflammation-mediated dry eye [32]. Interestingly, depression and inflammation are intertwined, mutually reinforcing and interdependent. Inflammation is the feature of many diseases and systemic disorders [33]. Therefore, choosing a drug which treats both depression and dry eye makes it easier for patient compliance. In this work, we induced mice to produce depression like behavior by CUMS procedure in a dry environment, and selected mice with dry eye state to explore the effect of luteolin on depression-related dry eye disorder and its potential mechanism.

Our experimental data showed that luteolin treatment significantly alleviated depression-like behaviors in mice, while the number of conjunctival epithelial cupped cells was restored and the symptoms of dry eye disease were significantly alleviated. Furthermore, we demonstrated that the effect of luteolin on dry eye depression co-morbidity was through modulating protein expression of key regulators in the Sirt1/NF-κB/ NLRP3 pathway. To prove this hypothesis, we used the Sirt1 inhibitor EX-527 to block Sirt1 protein expression. Unexpectedly, the therapeutic effect of luteolin did change, suggesting an important role of Sirt1 in luteolin’s alleviation of depression-like effects and dry eye disease symptoms.

Luteolin, a naturally occurring flavonoid, has been identified as one of the potential bioactive compounds with a variety of pharmacological effects. Research showed that luteolin could not only reduce the levels of pro-inflammatory factors TNF-α, IL-6, corticosterone, IL-17 and IL-23 [34], but also modulate Notch, FOXO, TNF, P53, Hippo, dopaminergic synapses and neurotrophin pathways [19]. Moreover, luteolin effectively reduced reactive oxygen species and thus improved nitric oxide utilization in venous endothelial cells [35]. Luteolin reduced the expression of cold-inducible RNA-binding protein (CIRP) mRNA and protein after cescal slurry injection (CSI). It was illustrated that luteolin decreased apoptosis, downregulated hypoxia-inducible factor-1α (HIF-1α) in septic neonates and LPS-stimulated neonatal macrophages [36]. Nevertheless, the pharmacological effect of luteolin on depression-related dry eye disorder had not been reported.

Luteolin reversed changes in levels of inflammatory and apoptosis-related proteins involving NF-κB, TNF-α, Sirt1, mTOR, p53, Bcl-2, and inhibited p38MAPK activation [37]. In our study, luteolin significantly reversed the symptoms of depression-related dry eye disorder, and we examined changes in the above proteins as well as inflammatory factors in both mouse serum and hippocampal tissues. Herein, we hypothesized that the effect of luteolin on the hippocampus through the blood-brain barrier was based on the Sirt1/NF-κB/NLRP3 signaling pathway. Genome-wide association studies have identified the genetic variants of Sirt1 associated with major depression [38]. Luteolin has been reported to exert therapeutic potential for age-related hearing loss by inhibiting H2O2-inducing cell senescence through the regulation of SIRT1 and p53 [39]. Luteolin also attenuated acute mercuric chloride induced hepatotoxicity by regulating Sirt1/Nrf2/TNF-α signaling pathway [37]. It has been demonstrated that the Sirt1 activity in mPFC pyramidal neurons plays a key role in the regulation of depression-related behaviors [40]. It had been reported that eIF2α deacetylation of lysine K143 by SIRT1 protects cardiac cells from endoplasmic reticulum stress, and it had also been shown that activation of Sirt1 protects cardiac cells from damage caused by endoplasmic reticulum stress [41]. Ning Jiang et al. demonstrated that Rg1 downregulates hippocampal neuroinflammation and upregulates hippocampal neurogenesis by regulating SIRT1 and decreasing the level of acetylated p65 (ac-p65) in the hippocampus, thus serving to ameliorate depression-like behavior and hippocampal neuroinflammation induced by chronic social defeat stress [42]. In addition, Sirt1 activation suppresses the protein level of NLRP3 inflammasome and also inhibits the expression of IL-1β, a marker of inflammasome [43]. The activation of the NLRP3 inflammasome requires ASC recruitment and automatic cleavage of Caspase-1 to Caspase-1, which promotes the activation of GSDMD and its cleavage to form GSDMD-N [44]. NLRP3 inflammasome assembles and causes pro-inflammatory programmed cell death, which is also known as cellular scorching [45]. It is widely believed that the transcription factor NF-κB drives the activation of NLRP3 inflammasomes during scorch death [46]. In the present study, after luteolin treatment, the protein levels of Ac-NF-κB, NF-κB, NLRP3, Cleaved-Caspase-1, Caspase-1, GSDMD and GSDMD-N were significantly downregulated in the hippocampus and cornea of mice compared with those of CUMS group, suggesting that luteolin might exhibit a beneficial effect on dry eye depression co-morbidity.

It has been shown that inhibition of Nlpr3 inflammatory vesicles by activation of Sirt1 acts on radiation-induced inflammatory bowel disease in mice [47]. In addition, it has been demonstrated that reducing pro-inflammatory cytokine levels by modulating Sirt1-NF-κB reverses depression-like behavior in olfactory bulbectomized rats [48]. Meanwhile, through our review of the literature, we learned that the SIRT1 inhibitor EX-527 attenuated depression-like behavior induced by CUMS stimulation [49]. To further validate the signaling pathway of luteolin for dry eye depression co-morbidity, we used the Sirt1 inhibitor EX-527 to inhibit Sirt1 protein expression levels, which reversed the ameliorative effects of luteolin on depression-like behavior, cognitive impairment, and dry eye disease symptoms in mice. The Ac-NF-κB, NF-κB, NLRP3, Cleaved-Caspase-1, Caspase-1, GSDMD and GSDMD-N related protein expression levels were also elevated by the application of EX-527. We confirmed that the Sirt1/NF-κB /NLRP3 signaling pathway was associated with luteolin treatment of depression-related dry eye disorder.

In summary, our experiments showed that luteolin could improve the symptoms of dry eye depression co-morbidities in mice through the signaling pathway Sirt1/NF-κB /NLRP3. Studies had shown that certain types of dry eye symptoms, especially those with increased frequency of blurred vision, are associated with depression and have a tendency to induce depression [50]. At the same time, we were concerned that in reports investigating the relationship between depression and xerophthalmia in the elderly, depression was associated with DED symptoms in subjects with normal or slightly reduced tear production [29]. Luteolin might be a candidate drug for the treatment of depression-related dry eye disorder, whereas further studies are needed before clinical application.

## MATERIALS AND METHODS

### Reagents

Luteolin (CAS: 491-70-3) was purchased from Shanghai McLean Biochemical Technology Co., Ltd. (L812409, Shanghai, China). Escitalopram (CAS: 128196-01-0) was purchased from Shanghai McLean Biochemical Technology Co., Ltd. (E883441, Shanghai, China). EX-527 (CAS: 49843-98-3) was provided by Sigma Biotechnology Co., Ltd. (E7034, Sigma-Aldrich, USA).

### Animals

Eight-week-old C57BL/6J male mice, weighing 18–22 g, were provided by Suzhou Spelford Biotechnology Co., Ltd (certificate number: SCXK(Jing)2019-0010). Animals were housed in normal-sized cages with free access to food and water under standard environmental conditions (22 ± 2°C, 12 h on/off light cycle, lights on at 8 am) and used for experiments after 3 days of acclimatization. All experimental procedures were approved by the Laboratory Animal Research Committee of Hunan University of Chinese Medicine and were performed in accordance with the Guide for the Care and Use of Laboratory Animals. The approval number of the ethics committee is No.2022-5. All procedures were in accordance with the institutional guidelines for the use of laboratory animals, and every effort was made to alleviate the suffering of laboratory animals.

### Groups and drug administration

According to the principle of random allocation, 40 mice were divided into five groups (8 mice in each group): control group, CUMS group, CUMS + Luteolin (5 mg/kg) group, CUMS + Luteolin (10 mg/kg) and CUMS + escitalopram group. Mariem Achour et al. [19] demonstrated that luteolin (10 mg/kg) reversed LPS-induced depression-like behaviors in mice. Luteolin (5 and 10 mg/kg) was used in this study. Luteolin and positive escitalopram (10 mg/kg) were dissolved in DMSO and normal saline. The concentration of DMSO was less than 0.1% (w/v). The drugs were orally treated once daily from 9:00 am to 10:00 am for 3 weeks.

### CUMS procedure and experimental design

To explore the effects of luteolin on depression-related dry eye disorder, we made some modifications to the CUMS procedure described earlier [51]. We eliminated the use of moist litter and forced swimming in mice, retaining stressors such as food and water deprivation, constant light, 45° cage tilt, restraint, flashing lights, tail pinching, and space crowding. Meanwhile, a fan was placed above the mouse cage, blowing at a moderate speed (about 600 r/min) for 12 h a day to keep the humidity in the cage below 30–35%. After 4 weeks of stress, luteolin (5 mg/kg), luteolin (10 mg/kg) and escitalopram (10 mg/kg) were administered by gavage once daily for three weeks. Meanwhile, the control group and the CUMS group were gavaged with normal saline. Mice were then sacrificed after assessing behavioral testing and dry eye testing. In Experiment 2, EX-527 (10 mg/kg) was administered intravenously for two consecutive weeks starting at the fifth week.

### Sucrose preference test (SPT)

The C57BL/6J mice undergoing the test were exposed to two bottles both containing 1% sucrose solution for 48 h to allow them to acclimatize, followed by 24 h of water and food deprivation. Subsequently, each mouse was exposed to two identical bottles (one containing pure water and the other containing 1% sucrose solution). After 6 h, the two bottle positions were swapped for 6 h and the bottles were weighed. The consumption of sucrose solution and pure water was determined by measuring the change in liquid consumption. Sucrose preference was defined as the ratio of sucrose volume to total sucrose volume and water consumption.

### Forced swimming test (FST)

The C57BL/6J mice were placed individually in a glass container (20 cm diameter and 45 cm high) for 6 min in water 15 cm deep at 25 ± 1°C. The last 4 min of each mouse was assessed by an investigator unaware of the groups. The immobility was defined as no movement and/or the small movement required to keep the head above the water surface. Each mouse was provided with fresh water.

### Tail suspension test (TST)

The C57BL/6J mice were individually suspended by tape approximately 20 cm from the ground and a transparent hollow anti-climbing tube (0.5 cm in diameter and 4 cm in length) was placed around their tails to prevent their tail-climbing behavior. The experiment lasted for 6 min and the time each mouse remained stationary (passively hanging and completely still) for the last 4 min was recorded and scored by a researcher who did not know the groups.

### Open field test (OFT)

The C57BL/6J mice were placed in a (100 × 100 × 40 cm) box made of opaque plexiglass. Each mouse was gently placed in the center of the apparatus during the test and allowed to explore freely for 5 min. Thereafter, the distance the mice walked and the time spent in the center were monitored by ANY-maze software. After each mouse was tested, the apparatus was washed with 75% ethanol to remove the odor left by the mice.

### Tear volume and corneal fluorescein staining

Tear secretion in mice was measured by the phenol red wire method at 9 am on day 28 and day 49, respectively. The silk threads were placed on the lateral eyes for 15 seconds, and the length of the wet red silk threads was recorded in millimeters. 5 μL (1%) of fluorescein dry was placed on the mouse eye surface to observe corneal epithelial disruption. Photographs of the eyes were taken with a digital camera. Each cornea was scored from 0 to 5 according to the Oxford University protocol.

### Nissl staining

Three mice from each group were selected for cardiac perfusion with 4% paraformaldehyde after behavioral testing. The removed brain tissue was fixed with 4% paraformaldehyde, embedded in paraffin, and sectioned. The slides were dehydrated in graded ethanol, soaked in xylene, and rehydrated in decreasing concentrations of ethanol. The slides were hydrated at 60°C for 30 min using 1% toluidine blue, dehydrated again with ethanol, xylene was removed, and finally sealed with neutral gel. The morphology of the hippocampus was observed under a light microscope.

### Western blot

The mouse hippocampus/cornea was extracted with RIPA lysis solution (containing 1% PMSF, 2% phosphatase inhibitor), and the mouse hippocampus/cornea protein was extracted, and centrifuged at 10,000 r/min at 4°C for 10 min. The protein concentration in the Western Blot samples was determined according to the instructions of the BCA protein concentration assay kit. Proteins were separated using 8–12% sodium dodecyl sulfate-polyacrylamide gels. The samples were electro transferred onto PVDF membranes and blocked with 5% milk solution. Membranes were then incubated overnight at 4°C with the following primary antibodies: anti-Sirt1 (CST, #8469, 1:1000), anti-NLRP3 (CST, #15101, 1:1000), anti-Cleaved-caspase-1 (CST, #89332S, 1:1000) anti-GSDMD-N (CST, #93709, 1:1000), anti-Caspase 1 (Abcam, ab207802, 1:1000). After washing 3 times, they were incubated with horseradish peroxidase-labeled secondary antibody for 1.5 h at room temperature, and protein bands were detected by enhanced chemiluminescence system and analyzed by ImageJ software.

### Conjunctival periodic acid-schiff (PAS) staining

4 μm sections were routinely dewaxed to water, then placed in 0.5% periodic acid oxidation solution at room temperature for 5–8 min, washed with tap water and twice with distilled water; the sections were soaked in Schiff reagent for 10–20 min at room temperature in a dark place, and rinsed with running water for 10 min; The sections were soaked in hematoxylin staining solution for 1 min, washed with tap water, differentiated with aqueous hydrochloric acid, washed with tap water, returned to blue with ammonia, and rinsed with running water. The sections were then placed in anhydrous ethanol I for 5 min, anhydrous ethanol II for 5 min, anhydrous ethanol III for 5 min, xylene for 5 min, and xylene II for 5 min. Consequently, the microscopic examination, image acquisition and analysis were conducted.

### Enzyme-linked immunosorbent assay (ELISA)

The Hippocampus/corneal homogenate was collected to be tested. The brain tissue was separated and homogenized, and the supernatant was harvested. The concentrations were determined by BCA commercial kit (Beyotime, Nanjing, China). The levels of TNF-α, IL-1β, IL-6, and IL-18 were detected by ELISA kit (Shanghai Zhuochai Biotechnology Co., Ltd, China) following the manufacturer’s instructions. The absorbance at recommended wavelength of each well was determined using Infnite F50 microplate reader (Tecan, Switzerland). The concentrations of cytokines were calculated from the standard curve.

### Immunofluorescence analysis

The paraffin sections of 4 μm brain tissue were routinely dewaxed to water, microwave heated with sodium citrate antigen repair solution for 10 min for antigen repair, washed with PBS, and sealed with 10% sheep serum (Solebo, SL038) at room temperature for 1 h. The blocking buffer was vacuumed, and diluted IBA1 primary antibody (1: 400, Wako, 019-19741) and rabbit anti-NLRP3 (PA579740, 1:1,000, Thermo Fisher, USA). Then the sample was incubated overnight at 4°C, and rinsed with 1× PBS for three times prior to the incubation with the second antibodies including Goat anti-rabbit IgG-H&L (Alexa Fluor^®^ 594)(1:1000, Abcam, ab150080) and Goat Anti-Rat IgG H&L (Alexa Fluor^®^ 488) (1:1000, Abcam, ab150165) at room temperature in dark for 1 h. Each sample was incubated with DAPI (Solebo, C0065) at dark for 10 min. After staining, the staining solution was removed and rinsed with 1× PBS for three times. The slides were sealed quenchable sealing tablets and observed under Fluorescence microscope.

### Molecular docking

The Sirt1 (PDB code:5BTR) and Luteolin (chemical book: #491-70-3)/Resveratrol (chemical book: #501-36-0) was prepared by Discovery Studio. The combining site was defined according to endogenous ligand. The protein ligand interaction was calculated using CDOCKER method. The interaction and CDOCKER ENERGY were observed.

### Statistical analysis

The experimental data were expressed as Mean ± SEM. Statistical analysis was performed using one-way analysis of variance (ANOVA) and Dunnett’s multiple comparisons test with GraphPad prism 7 software, ^*^p < 0.05 represents statistical difference.
